# Solidarity in the critical zone: habitability and the reconstruction of the sociological concept of solidarity

**DOI:** 10.3389/fsoc.2026.1800408

**Published:** 2026-09-29

**Authors:** Marco Bontempi

**Affiliations:** Department of Political and Social Sciences, University of Florence, Florence, Italy

**Keywords:** Bruno Latour, climate change, climate justice, climate solidarity, critical zone, habitability, social solidarity, sociological theory

## Abstract

Climate change is transforming not only the empirical conditions within which solidarity operates but also the sociological explanandum that the concept of solidarity has traditionally been called upon to explain. While classical sociology conceptualised solidarity primarily as the organisational principle through which differentiated societies maintain collective continuity, contemporary climate scholarship increasingly addresses the material conditions upon which collective existence itself depends. Despite significant advances in Climate Justice, Climate Solidarity, Commons and Commoning, Political Ecology, and Earth System Governance, these perspectives reconstruct different organisational dimensions of this transformation without providing a common sociological explanation of the changing explanandum of solidarity. This article aims to reconstruct the sociological concept of solidarity by proposing terrestrial solidarity as a framework centred on the collective organisation of participation in the co-production of habitability. Methodologically, the article adopts a strategy of theoretical reconstruction rather than theory testing or comparative theory-building. It first reconstructs five contemporary theoretical perspectives as complementary responses to an emerging explanatory problem. Building on Bruno Latour’s concepts of the Critical Zone, actants, mediation, Gaia, autotrophy and heterarchy, it then develops a conceptual framework that redefines solidarity in relation to habitability. Finally, the framework is examined through three heterogeneous empirical configurations—the Rhine River basin, the Danube River basin and Community-Supported Agriculture—not as empirical tests but as reconstructive settings through which complementary organisational operations become analytically observable. The article argues that the explanandum of solidarity has shifted from the reproduction of society to the collective organisation of participation in sustaining the conditions of collective existence. It proposes terrestrial solidarity as a sociological framework that reconstructs how responsibility, durable commitments, collective maintenance, socio-ecological co-production and institutional coordination become complementary organisational dimensions of the same explanatory architecture. In doing so, the article offers a common sociological framework through which dispersed contributions from contemporary climate scholarship become theoretically intelligible while redefining solidarity as the collective organisation of participation in sustaining habitability.

## 1 Introduction

Few concepts have occupied a more enduring place within sociological theory than solidarity because few concepts have been so closely associated with one of sociology’s foundational explanatory questions: how collective existence becomes possible under conditions of increasing differentiation. From its classical formulations onwards, the sociological concept of solidarity has not primarily referred to moral dispositions, altruistic behaviour or voluntary cooperation among individuals. Rather, it has functioned as an explanatory concept through which sociology has reconstructed the organisational conditions that enable collective life to persist despite differentiation, complexity and social change.

This distinction is important because the sociological concept of solidarity should not be conflated with solidarity as an empirical phenomenon. Acts of cooperation, mutual support or collective commitment constitute observable social practices. The sociological concept of solidarity, by contrast, is an explanatory instrument through which such practices acquire theoretical significance. Concepts explain; they are not themselves the phenomena they explain. This distinction becomes decisive once climate change begins to transform what solidarity is expected to explain. Throughout the history of sociological theory, different conceptions of solidarity have therefore addressed a common explanatory object: how collective existence maintains its continuity under changing conditions of social differentiation. The argument developed in this article is not that solidarity as an empirical phenomenon has fundamentally changed. Rather, it asks whether what the sociological concept of solidarity has traditionally been called upon to explain has itself undergone a profound transformation.

Climate change raises precisely this possibility. Contemporary environmental transformations do not simply introduce new empirical problems to which existing theories of solidarity may subsequently be applied. They call into question the explanatory object around which the sociological concept of solidarity has historically been organised. As ecological degradation, biodiversity loss, hydrological instability and climate disruption increasingly reshape the material conditions of collective existence, the question is no longer confined to how societies maintain integration under conditions of differentiation. It increasingly concerns how heterogeneous human and non-human processes collectively participate in maintaining, transforming and reproducing the conditions upon which collective existence itself depends (Rockström et al., 2009; Steffen et al., 2015; Lenton, 2016).

This shift should not be understood as replacing the classical sociological problem with an environmental one. Rather, it suggests that the conditions through which collective life becomes possible can no longer be reconstructed exclusively within the boundaries of society conceived as an autonomous social order. The material processes that sustain collective existence increasingly become inseparable from the conditions that the sociological concept of solidarity is called upon to explain. Consequently, the challenge raised by climate change does not consist simply in extending existing theories of solidarity to environmental issues. It consists in asking whether the explanatory object of the sociological concept of solidarity has itself been transformed (Chakrabarty, 2009; Latour, 2017).

The distinction between the sociological concept of solidarity and its explanandum is therefore central to the argument developed in this article. By *explanandum* we do not mean the sociological concept of solidarity itself, but the phenomenon that the concept is intended to explain. This distinction allows us to separate changes in the empirical manifestations of solidarity from changes in the explanatory task assigned to the sociological concept of solidarity. The argument developed here is not that climate change simply generates new forms of solidaristic practice. Rather, it transforms what the sociological concept of solidarity has traditionally been called upon to explain, thereby requiring a corresponding reconstruction of the concept itself.

Although contemporary scholarship has developed a wide range of responses to climate change, these responses increasingly converge around a common intuition. Climate change is no longer understood solely as an environmental problem requiring new forms of political cooperation or ethical commitment. It has progressively become a challenge to the conditions through which collective existence is organised and sustained. As a consequence, the sociological concept of solidarity has itself begun to evolve beyond its classical concern with social integration alone (Tschakert et al., 2021).

This transformation is visible across several contemporary bodies of literature. Although these perspectives differ substantially, they share one important characteristic: each addresses a different dimension of the changing relationship between collective organisation and the conditions of collective existence. Climate Justice has demonstrated that environmental transformations redistribute vulnerabilities, responsibilities and capacities across societies in profoundly unequal ways (Schlosberg, 2007; Schlosberg and Collins, 2014; Newell et al., 2021). Climate Solidarity has examined how durable commitments can be maintained under conditions of uncertainty, temporal asymmetry and differentiated exposure to climate risks (Tschakert et al., 2021). Commons and Commoning have shifted attention towards the collective maintenance of shared conditions of reproduction rather than their mere distribution (Ostrom, 1990; Bollier and Helfrich, 2019). Political Ecology has shown that these conditions emerge through heterogeneous socio-ecological relations rather than existing as external constraints upon society (Robbins, 2020; Escobar, 2018). Earth System Governance has reconstructed the institutional coordination required when ecological processes exceed the capacities of individual political jurisdictions (Biermann et al., 2009; Biermann, 2014; Young, 2017).

Taken together, these perspectives suggest that the sociological problem of solidarity is gradually expanding beyond the organisation of social relations alone. Yet they do not converge around a common explanatory object. Each reconstructs a different organisational dimension of collective existence because each addresses a different theoretical problem. Their analytical diversity should therefore not be understood as theoretical fragmentation, but as evidence that the relationship between solidarity and the conditions of collective existence is itself being reformulated. What remains unclear, however, is whether these parallel developments point towards a more fundamental transformation in what the sociological concept of solidarity is called upon to explain.

It is precisely this question that motivates the present article.

This article argues that the developments described above cannot be fully understood simply as the emergence of new theories of solidarity. Rather, they indicate that what the sociological concept of solidarity is called upon to explain has itself been transformed. If this is the case, extending existing theories of solidarity to climate change is not sufficient. A corresponding reconstruction of the sociological concept of solidarity itself becomes necessary.

The article develops this argument in three steps. First, it reconstructs how contemporary climate-related scholarship progressively reorients the explanatory object of solidarity beyond the classical concern with the reproduction of society alone. Rather than treating Climate Justice, Climate Solidarity, Commons and Commoning, Political Ecology and Earth System Governance as competing theoretical perspectives, it reconstructs them as complementary responses to different organisational dimensions of an emerging explanatory problem.

Second, the article proposes the concept of terrestrial solidarity as a sociological reconstruction capable of bringing these dispersed organisational functions into a common explanatory framework. Terrestrial solidarity is not introduced as an additional normative model of solidarity, nor as an alternative to existing approaches. It is proposed as a sociological concept through which the collective organisation of participation in the co-production of habitability becomes theoretically intelligible.

Third, the article examines three heterogeneous empirical configurations—the Rhine River basin, the Danube River basin and community-supported agriculture—not as empirical tests of the proposed framework, but as configurations through which its theoretical operations become analytically observable. Their role is reconstructive rather than illustrative or verificatory: they render visible complementary organisational processes that the theoretical framework subsequently recomposes within a single sociological explanation.

Taken together, these three steps seek to show that climate change does not simply require new forms of collective action. It requires a reconstruction of the sociological concept of solidarity adequate to conditions in which collective existence increasingly depends upon the organised participation of heterogeneous human and non-human processes in the co-production of habitability.

The argument therefore follows a progressive reconstruction: from the classical sociological problem, through the theoretical reformulation proposed in this article, to the empirical reconstruction of its organisational functions, before recomposing them within a single sociological explanation and reflecting on their broader implications for contemporary sociological theory.

## 2 Literature review

### 2.1 The classical sociological problem of solidarity

Solidarity has occupied a foundational place in sociological theory because it addresses one of sociology’s central explanatory questions: how collective order becomes possible under conditions of social differentiation. Rather than being understood primarily as a moral disposition, an ethical ideal or a form of altruistic commitment, solidarity has traditionally functioned as an explanatory concept. Across otherwise divergent theoretical traditions, it has provided the answer to a common sociological problem: how differentiated individuals, groups and institutions become part of a collective order capable of maintaining its continuity over time.

This explanatory orientation receives its classical formulation in *The Division of Labour in Society*. Durkheim’s distinction between mechanical and organic solidarity does not primarily describe two moral attitudes but two mechanisms through which collective cohesion is generated under different conditions of social differentiation. Mechanical solidarity is grounded in similarity and the predominance of a common collective consciousness, whereas organic solidarity emerges from the increasing interdependence produced by the division of labour. Despite these differences, both forms of solidarity explain the same phenomenon: how society reproduces itself as a coherent collective order despite growing internal differentiation (Durkheim, 1893/1984).

Subsequent sociological traditions substantially reformulated the mechanisms of integration while preserving this explanatory orientation. Parsons translated the Durkheimian problem into a theory of systemic integration, in which differentiated institutions contribute to the continuity of the social system through normative coordination (Parsons, 1951). Habermas (1984, 1987), in turn, rejected functionalist reductionism by locating solidarity in processes of communicative action through which actors reproduce shared meanings, legitimate social relations and collective identities. Although these perspectives differ profoundly in their understanding of social order, they remain organised around the same analytical question: how society maintains its continuity despite increasing differentiation and complexity.

The differences among these traditions should therefore not obscure their deeper theoretical convergence. Durkheimian moral integration, Parsonian systemic integration and Habermasian communicative integration identify different mechanisms through which collective order is reproduced, yet they continue to treat society itself as the primary object requiring explanation. The material conditions of collective existence undoubtedly influence social reproduction, but they remain analytically external to the solidaristic mechanisms through which society is constituted and maintained. They appear as conditions of social life, not as constitutive participants in the production of solidarity itself. What unites these traditions is therefore not a common theory of solidarity, but a common explanatory problem (Alexander, 1982–1983).

Recovering this classical architecture is not intended to expose an ecological omission retrospectively. Classical sociology addressed a historically specific problem: how modern societies achieve and reproduce collective integration under conditions of differentiation. The challenge raised by climate change is therefore not simply to extend existing theories of solidarity to environmental issues, but to ask whether what the sociological concept of solidarity has traditionally been called upon to explain has itself been transformed (Chakrabarty, 2009; Latour, 2017).

### 2.2 Climate change and the transformation of the explanatory problem

If classical sociology treated solidarity as the mechanism through which society reproduces itself, climate change calls attention to a different question: how the material conditions of collective existence are themselves produced, maintained and transformed. This shift does not simply add environmental concerns to an otherwise unchanged sociological agenda. Rather, it challenges the assumption that the continuity of society can be analytically separated from the processes through which the conditions enabling collective life are continuously sustained.

Developments in Earth System Science have progressively reconstructed the Earth as an integrated system whose long-term habitability emerges from interacting biophysical processes operating across multiple temporal and spatial scales (Rockström et al., 2009; Steffen et al., 2015; Lenton, 2016, 2025). Rather than treating environmental degradation as a collection of external pressures acting upon society, this perspective understands the Earth as a dynamic system whose relative stability results from the continuous interaction of climatic, geological, chemical and biological processes. Although these approaches do not propose a sociological theory of solidarity, they fundamentally transform the status of the material conditions upon which collective life depends. Climate, freshwater systems, soils, biodiversity and biogeochemical cycles can no longer be treated simply as a stable background to social action. Human societies increasingly participate in transforming the very Earth-system processes through which habitable conditions are continuously maintained (Lenton, 2016; Lenton, 2025).

The implications of this transformation extend well beyond Earth System Science itself. Chakrabarty argues that climate change unsettles the conventional separation between human history and Earth history because human beings now appear simultaneously as historically differentiated political actors and as a force capable of altering planetary processes (Chakrabarty, 2009). The significance of this argument lies less in redefining humanity than in revealing that the object requiring sociological explanation can no longer be confined to the internal reproduction of society alone. The conditions supporting collective existence have themselves become historically dynamic and increasingly entangled with human action.

Within sociology and political theory, this epistemological shift has been developed most systematically by Latour. He argues that the ecological crisis cannot be understood simply by extending existing social categories to environmental issues, but requires rethinking the entities, relations and collectives through which political and sociological analysis is organised (Latour, 2014, 2017, 2018; Latour and Weibel, 2020; Lenton and Latour, 2018). The importance of this contribution lies not in providing a new sociological theory of solidarity, but in demonstrating that the transformation disclosed by Earth System Science also requires a corresponding transformation in the conceptual categories through which collective existence is analysed.

Yet recognising this transformation does not imply abandoning historical and political differentiation. Bonneuil and Fressoz (2016) warn against narratives in which an undifferentiated humanity emerges as the collective subject of the Anthropocene, obscuring the unequal histories of industrialisation, colonialism, fossil-fuel dependence and political responsibility that have produced contemporary ecological disruption. The material conditions upon which collective existence depends are increasingly subject to collective human transformation, but the capacities to transform them, the benefits derived from their exploitation and the vulnerabilities associated with their degradation remain profoundly unequal. Any sociological reformulation of solidarity must therefore preserve constitutive interdependence without erasing differentiated responsibility.

Climate change therefore transforms not simply the empirical conditions addressed by sociology, but the explanatory problem around which the sociological concept of solidarity has historically been organised. The challenge is no longer simply to explain how societies reproduce themselves, but to understand how contemporary theory has responded to the altered relationship between collective organisation and the material conditions of collective existence. This transformation, in turn, raises a further question: which organisational dimensions have contemporary theories reconstructed in response to this new explanatory problem, and how might these dispersed contributions be recomposed within a common sociological framework?

#### 2.2.1 Climate justice

Among contemporary approaches, Climate Justice has made one of the most significant contributions to this theoretical reorientation by demonstrating that the disruption of the material conditions of collective existence cannot be understood independently of questions of historical responsibility, unequal vulnerability and distributive justice (Schlosberg, 2007; Schlosberg and Collins, 2014; Newell et al., 2021). It provides an appropriate point of departure because it was among the first approaches to recognise that climate change transforms not only environmental conditions but also the distribution of responsibilities through which collective existence is sustained.

Climate Justice shifts the analytical focus from environmental degradation as a generic global problem to the differentiated social relations through which climate change is produced, experienced and addressed. Building on principles of distributive, procedural, recognitional and restorative justice, this perspective has shown that climate impacts and adaptive capacities are unevenly distributed across countries, communities and social groups, reflecting long-standing asymmetries of wealth, political power and historical responsibility (Schlosberg, 2007; Newell et al., 2021). This analytical reorientation is particularly visible in debates surrounding *loss and damage*, which recognise that climate change generates obligations that cannot be reduced either to humanitarian assistance or to market-based compensation alone (Calliari et al., 2020; Roberts and Pelling, 2018).

The principal contribution of Climate Justice therefore lies in demonstrating that the conditions upon which collective existence depends are neither politically neutral nor socially homogeneous. They are shaped through historically differentiated relations of production, extraction, consumption and exposure to environmental risk. Any attempt to rethink solidarity under conditions of climate change must therefore incorporate differentiated responsibilities and unequal capacities for action as constitutive dimensions rather than secondary normative considerations.

Climate Justice therefore reconstructs the normative architecture of differentiated responsibility without yet fully theorising the organisational conditions through which the continuity of collective existence is maintained. It renders the transformed explanatory problem clearly visible while leaving open the question of how the collective organisation of the conditions of habitability is itself to be conceptualised.

#### 2.2.2 Climate solidarity

Among the contemporary theoretical responses reviewed here, Climate Solidarity constitutes the perspective most closely aligned with the concerns developed in this article because it places solidarity itself—not only justice, governance or environmental management—at the centre of climate transformation (Tschakert et al., 2021; Bazzani, 2023). Rather than treating solidarity as a secondary normative consequence of climate change, this literature reconstructs it as a constitutive condition for sustaining collective responses to an increasingly unstable world. In doing so, it reopens solidarity as a central sociological category for understanding how durable commitments, mutual support and shared responsibilities can be organised under conditions of ecological disruption.

In doing so, Climate Solidarity complements the distributive emphasis of Climate Justice by asking how durable collective commitments can be sustained over time. Its principal contribution lies in demonstrating that climate change cannot be addressed solely through distributive principles or institutional coordination. Collective capacities to respond depend equally upon the construction of solidaristic relations capable of sustaining cooperation across differences, maintaining commitments over time and institutionalising reciprocal obligations despite uncertainty and unequal vulnerability (Tschakert et al., 2021; Bazzani, 2023; Bazzani, 2024). In this perspective, solidarity is understood not as spontaneous altruism but as an organised and durable social arrangement through which pro-social commitments become stabilised over time, thereby enabling collective action to endure across conditions of uncertainty (Bazzani, 2024).

The present framework builds directly upon this theoretical achievement. Climate Solidarity succeeds in moving solidarity from the margins to the centre of climate sociology, thereby bringing the emerging explanatory problem substantially closer to sociological analysis. At the same time, its analytical centre of gravity remains primarily located in the organisation of commitments, responsibilities and reciprocal obligations among human actors.

The perspective developed here proposes a complementary shift. Rather than asking only how solidaristic commitments among human actors can be organised, it asks how collective organisation participates in composing, maintaining and transforming the material conditions upon which collective existence depends. This does not replace solidaristic relations with ecological processes; rather, it situates solidaristic relations within the broader organisation of the material conditions that sustain collective existence.

Climate Solidarity therefore reconstructs the organisational conditions through which durable commitments become possible under conditions of ecological disruption. It thereby brings the transformed explanatory problem substantially closer to sociology while leaving open a further question: how are these solidaristic commitments related to the material conditions through which collective existence itself is sustained? Recent work has further extended this research agenda by situating climate solidarity within a broader theory of altruistic solidarity capable of addressing multiple global collective challenges (Bazzani, 2025).

#### 2.2.3 Commons and commoning

The commons and commoning perspective extends this trajectory by shifting analytical attention from solidaristic commitments towards the collective organisation of the material conditions upon which shared life depends. Rather than treating collective action primarily as the coordination of obligations among actors, research on commons and commoning demonstrates how the conditions sustaining collective life themselves become objects of shared organisation, maintenance and reproduction (Ostrom, 1990; Bollier and Helfrich, 2019). In doing so, it complements Climate Solidarity by asking not only how commitments are sustained, but how the material conditions that make those commitments possible are themselves collectively maintained.

Beginning with Ostrom’s institutional analysis of common-pool resources and subsequently expanded through the literature on commoning, this perspective progressively displaced attention from resources understood as pre-given objects towards the collective practices through which they are constituted, governed and reproduced (Ostrom, 1990; Bollier and Helfrich, 2019). Commons are therefore not simply material goods requiring protection, but ongoing socio-material achievements sustained through shared rules, reciprocal commitments and continuous collective work. This shift represents a significant theoretical advance because it relocates the focus of collective organisation from solidaristic relations alone to the conditions through which those relations can endure over time.

The contribution of this perspective becomes particularly evident in practices such as community-supported agriculture (CSA), where collective organisation extends beyond the distribution of agricultural products to encompass the shared maintenance of productive conditions, the redistribution of risks and the long-term reproduction of socio-ecological relationships (European CSA Research Group, 2016; Bollier and Helfrich, 2019; Vivero-Pol et al., 2019). In these experiences, cooperation is sustained through the collective maintenance of the material conditions upon which it depends.

The Commons and Commoning perspective therefore reconstructs the organisational conditions through which the material bases of collective existence become objects of shared maintenance and reproduction. For the first time within the trajectory reconstructed here, these conditions become explicit objects of collective organisation rather than remaining the implicit background of solidaristic commitments.

At the same time, its analytical focus remains directed primarily towards the governance and reproduction of particular commons rather than towards the broader socio-ecological processes through which the conditions of collective existence are continuously produced. It therefore leaves open a further question: how are these conditions themselves generated through heterogeneous relations linking human and non-human processes?

#### 2.2.4 Political ecology

The Political Ecology perspective extends this trajectory by shifting analytical attention from the collective organisation of shared conditions to the socio-ecological processes through which those conditions are continuously produced and transformed. In doing so, it complements the Commons and Commoning perspective by asking not only how shared conditions are collectively maintained, but how those conditions themselves are continually generated through heterogeneous socio-ecological relations. Rather than treating environmental conditions as stable resources requiring collective management, Political Ecology reconstructs them as relational outcomes emerging from historically situated interactions among ecological processes, economic practices, technological infrastructures and political power (Robbins, 2020; Escobar, 2018).

Its principal contribution lies in demonstrating that the conditions sustaining collective existence cannot be understood independently of the heterogeneous relations through which they are continually produced. Climate change, environmental degradation and ecological vulnerability therefore appear not as external disturbances affecting otherwise stable societies, but as outcomes of socio-ecological configurations structured through unequal distributions of power, labour, metabolism and access to environmental resources (Robbins, 2020; Escobar, 2018; Swyngedouw, 2004). In doing so, Political Ecology substantially deepens this reorientation by relocating analytical attention from collectively organised conditions to the relational processes through which those conditions themselves are constituted.

This relational perspective represents a decisive theoretical advance. The analytical object is no longer the governance of particular commons but the heterogeneous socio-ecological relations through which conditions of collective existence are continuously generated, maintained and transformed. Material conditions no longer appear as the background of collective action nor simply as objects of collective organisation, but as dynamic configurations emerging through ongoing processes of production and transformation.

Political Ecology therefore reconstructs the relational processes through which the material conditions of collective existence are continuously produced. At the same time, its analytical emphasis remains directed primarily towards explaining socio-ecological production rather than towards reconstructing the organisational forms through which these heterogeneous relations acquire continuity, durability and coordination over time. It therefore leaves open a further question: how do these distributed socio-ecological processes become collectively coordinated across multiple institutional scales and temporal horizons?

### 2.3 Earth system governance

The Earth System Governance perspective extends this trajectory by shifting analytical attention from the production of socio-ecological conditions to the institutional configurations through which distributed socio-ecological processes can be coordinated across multiple spatial and temporal scales. In doing so, it complements Political Ecology by asking not only how socio-ecological conditions are produced, but how these distributed processes become institutionally coordinated without reducing their complexity. Rather than treating governance as the administration of discrete environmental problems, this perspective reconstructs climate change as a challenge of organising complex socio-ecological transformations under conditions of deep interdependence, uncertainty and institutional plurality (Biermann et al., 2009; Biermann, 2014).

Its principal contribution lies in demonstrating that the conditions sustaining collective existence cannot be addressed through isolated institutions or single-level political authority. Instead, governance emerges through adaptive, polycentric and multi-level arrangements capable of coordinating heterogeneous actors, forms of knowledge and decision-making processes operating across interconnected ecological and social systems (Folke et al., 2005; Gupta et al., 2010; Young, 2017; Dryzek, 2016). The analytical object consequently shifts once again: attention is directed not simply towards the production of socio-ecological relations but towards the institutional configurations through which distributed processes acquire continuity, coherence and adaptive capacity across multiple scales.

This perspective substantially enriches the emerging account by recognising that the continuity of collective existence depends not only upon the production of socio-ecological conditions but also upon institutional capacities to coordinate those processes across jurisdictions, temporal horizons and domains of collective action (Biermann, 2014; Young, 2017). Governance is therefore understood less as the exercise of centralised control than as the continual organisation of adaptive coordination within complex and evolving socio-ecological configurations. In doing so, Earth System Governance demonstrates that collective existence increasingly depends upon institutional arrangements capable of responding to complexity without reducing it.

Earth System Governance therefore reconstructs the institutional conditions through which distributed socio-ecological processes become collectively coordinated across multiple scales. In doing so, it brings the trajectory reconstructed throughout this review to its furthest point. At the same time, its analytical focus remains directed primarily towards the coordination of those processes rather than towards reconstructing the collective organisation of the material conditions whose continuity such coordination ultimately seeks to sustain. The question that consequently remains open is not simply how distributed socio-ecological processes can be coordinated, but how the material conditions upon which collective existence depends can themselves become the collective object around which solidarity is organised.

Taken together, these perspectives reveal a remarkable transformation in contemporary sociological and interdisciplinary thinking about climate change (Chakrabarty, 2009; Latour, 2017). Rather than constituting competing explanatory paradigms, they progressively reconstruct the explanatory problem addressed by the sociological concept of solidarity, relocating the analytical centre of gravity from the reproduction of society towards the organisation of the material conditions upon which collective existence depends. Climate Justice reconstructs differentiated responsibilities; Climate Solidarity explains how pro-social commitments become organised and stabilised over time; Commons and Commoning relocate analytical attention towards the collective maintenance of shared conditions; Political Ecology demonstrates that these conditions are continuously produced through heterogeneous socio-ecological relations; Earth System Governance shows how these distributed processes require adaptive forms of institutional coordination across multiple scales.

Considered together, these perspectives do not simply offer alternative explanations of climate change. They document a cumulative sequence of theoretical operations through which the explanatory problem progressively changes. Each framework renders visible a previously underdeveloped organisational dimension while simultaneously leaving open the question that motivates the next theoretical move.

The following theoretical framework does not introduce an alternative paradigm but develops this trajectory by reconstructing the organisational architecture through which these dispersed theoretical operations become mutually intelligible within a common sociological explanation. It proposes terrestrial solidarity as a sociological framework capable of explaining how the material conditions upon which collective existence depends become the collective object around which solidarity is organised.

## 3 Theoretical framework

The preceding literature review has shown that contemporary climate scholarship progressively reconstructs a new explanatory problem without yet providing a common sociological framework through which it can be understood. The question therefore becomes how sociology can account for collective existence once habitability becomes what the sociological concept of solidarity is called upon to explain.

Existing perspectives successfully reconstruct responsibility, governance, commoning, socio-ecological production and collective commitment. None of them, however, was developed to conceptualise habitability as the collective object around which solidarity itself must be reconstructed. Once habitability becomes the explanatory problem, the analytical focus shifts from the regulation of pre-existing conditions to the processes through which those conditions are continuously composed, maintained and transformed. This shift requires an ontology capable of understanding the conditions of collective existence not as external constraints or stable environmental contexts, but as ongoing relational achievements.

Among the available conceptual resources, Bruno Latour’s work on the Critical Zone provides precisely such an ontology (Latour, 2017; Latour and Weibel, 2020; Arènes et al., 2018). Rather than treating the Earth as a global object or as a passive environmental backdrop, it redirects analytical attention to the thin, heterogeneous layer of the planet within which the conditions of habitability are continuously produced and maintained. In Earth System Science, the Critical Zone designates the narrow surface layer extending from the lower atmosphere through vegetation, soils and active groundwater to the underlying weathered rock, where physical, chemical and biological processes become inseparably intertwined in sustaining life (Lenton, 2016; Lenton, 2025).

Latour’s contribution does not consist simply in adopting this scientific concept as a descriptive background. Rather, he reconstructs the Critical Zone as the relational domain within which the conditions of habitability emerge through the continuous composition of heterogeneous entities whose interactions participate in maintaining, transforming and, at times, destabilising those conditions (Latour, 2017; Latour and Weibel, 2020). The significance of this reformulation lies in the fact that it moves beyond approaches explaining collective existence exclusively through interactions among human actors. Instead, it opens an analytical space in which participation in the composition of habitability is no longer restricted to human actors alone.

Once habitability is understood as a relational achievement, however, a further question immediately arises: who—or what—participates in its composition?

Once habitability is understood as emerging through the continuous composition of heterogeneous relations, restricting analytical participation to human actors alone becomes insufficient. The processes through which habitability is maintained involve heterogeneous entities whose effects cannot be understood independently of the relations in which they participate. Following Latour (2005), these participants are referred to as actants.

The significance of this concept does not lie in attributing equivalent forms of agency to all entities. Rather, it shifts analytical attention from the intrinsic properties of entities to the relational processes through which they participate in composing, maintaining or transforming the conditions of habitability. Whether biological, geological, hydrological, technological, institutional or legal, these heterogeneous entities become analytically relevant insofar as they contribute, in different ways and to different degrees, to the ongoing composition of habitability.

The concept of the actant therefore designates neither a new category of beings nor a simple extension of the sociological category of actor. It identifies a relational position within the processes through which collective existence is continuously composed. The analytical question consequently shifts from asking what an entity is to asking how it participates in the relational composition of habitability.

Recognising heterogeneous entities as actants immediately raises a further question: if participation is distributed across such different entities, how can their contributions become organised into coherent courses of action rather than remaining a mere aggregation of heterogeneous effects?

Latour’s answer does not consist in attributing agency as an intrinsic property possessed by either human or non-human entities. Rather, agency emerges through the mediated associations that connect heterogeneous participants within ongoing courses of action (Latour, 1996; Latour, 2005).

From this perspective, action is neither produced by isolated actors nor simply distributed across multiple entities. It is continuously translated, redirected and transformed through the relations that connect them. Agency therefore appears less as a property of individual participants than as a relational accomplishment emerging through processes of mediation.

This reformulation fundamentally changes the sociological question. Explaining collective existence no longer means identifying who acts, but reconstructing how action circulates through relational configurations that continuously compose, maintain or transform the conditions of habitability. Mediation is therefore not an intermediary stage between already constituted actors; it is the relational process through which heterogeneous participants become capable of jointly contributing to the ongoing composition of habitability.

If action is continuously mediated across heterogeneous participants, however, a further question follows: how can such relational configurations persist over time rather than remaining episodic and unstable?

Latour addresses this question through his reinterpretation of Gaia (Latour, 2017; Lenton et al., 2018; Lenton et al., 2020).

Rather than conceiving Gaia as a superorganism, a self-regulating system or an overarching entity standing above its components, he reconstructs it as the historically evolving composition of the conditions that sustain life within the Critical Zone (Latour, 2017; Lenton, 2025). Gaia therefore does not designate a unified whole existing beyond its constituent processes, but the continuous historical composition through which heterogeneous relations collectively maintain, transform and sometimes destabilise the conditions of habitability.

What distinguishes living processes is not simply that they influence environmental conditions. Their distinctive contribution lies in participating in feedbacks through which the conditions of their own habitability are continuously modified, redistributed and partially stabilised (Lenton et al., 2020; Lenton, 2025). Life does not merely adapt to external environmental conditions; through its own metabolic activity it contributes to shaping those very conditions while remaining continuously exposed to the consequences of its own transformations.

From this perspective, Gaia no longer appears as a stable equilibrium or as a system maintaining itself from above. Rather, it designates the historically contingent composition of intertwined feedbacks through which habitability is continuously reproduced, transformed and rendered vulnerable to its own dynamics. Historical continuity therefore becomes an emergent achievement of ongoing relational composition rather than the expression of a pre-existing order.

This reformulation nevertheless raises a further question: if habitability persists through recursive feedbacks, how do living processes continuously contribute to producing the very conditions upon which their own persistence depends?

Latour, drawing on Earth System Science, addresses this question through the concept of autotrophy (Lenton et al., 2020; Lenton, 2025).

Here, autotrophy is not understood simply as a biological classification distinguishing particular organisms from heterotrophic life. Rather, it identifies a more fundamental generative principle: living processes participate in producing the material conditions that make their own persistence possible (Lenton et al., 2020; Lenton, 2025). Life does not merely occupy a pre-existing environment or adapt to external constraints. Through its metabolic activity, it continuously contributes to producing, maintaining and transforming the very conditions upon which it depends.

This capacity does not distinguish particular kinds of entities but a distinctive mode of participation in the composition of habitability. While both living and abiotic processes participate in shaping environmental conditions, only living processes contribute to producing the conditions upon which their own persistence depends and are recursively exposed to the consequences of their own contributions. Autotrophy therefore identifies the recursive generativity through which life participates in the historical continuity of habitability.

From this perspective, autotrophy no longer designates merely a biological property. It becomes the principle through which the ongoing reproduction of habitability can be understood as an internally generated process rather than as adaptation to externally given environmental conditions. The continuity of habitability consequently appears not as the persistence of a stable environment but as the recursive regeneration of the conditions upon which life itself continuously depends.

Yet recursive generation alone cannot explain how innumerable living and non-living processes operating simultaneously across different spatial and temporal scales remain collectively compatible over time. A further question therefore arises: how can distributed recursive processes maintain organisational coherence without being coordinated by a single organising centre?

Building on Latour’s relational ontology, the concept of heterarchy offers a way of approaching this problem.

Rather than describing a decentralised institutional arrangement, heterarchy identifies a relational mode of organisation in which multiple mechanisms of coordination operate simultaneously across heterogeneous processes and scales. Stability is therefore neither imposed from above nor produced through spontaneous equilibrium. Instead, it emerges through the continuous compatibility of partially overlapping relational adjustments distributed across heterogeneous processes operating according to different temporal rhythms and spatial scales.

From this perspective, heterarchy does not explain the production of habitability itself—that remains the distinctive contribution of autotrophic processes. Rather, it explains the organisational conditions through which recursive processes remain mutually compatible while continuously reorganising in response to changing circumstances. Organisational coherence consequently appears not as the expression of hierarchical control but as the emergent outcome of distributed processes of coordination that continually accommodate one another without converging upon a single organising principle.

Heterarchy therefore provides the organisational principle through which the historical continuity of habitability can emerge from distributed processes of composition without presupposing either hierarchical control or a single integrating centre. It explains how heterogeneous recursive processes remain organisationally compatible while continuously transforming the conditions under which they operate.

Up to this point, the framework explains how habitability is composed, historically reproduced and organisationally sustained. It does not yet explain how human societies organise their own participation within these processes. Material interdependence alone does not generate solidarity, nor do distributed relations of dependence by themselves produce collective commitments to sustain the conditions upon which collective existence depends. A further conceptual step is therefore required. The following section develops this step through the concept of terrestrial solidarity.

We propose terrestrial solidarity as the collective organisation of reflexive obligations through which human actors intentionally coordinate their participation in sustaining habitability. Terrestrial solidarity is therefore not grounded primarily in shared identity, reciprocity or moral obligation alone. It emerges from the recognition that habitability itself constitutes the common condition of existence whose continued reproduction depends upon organised forms of collective action (Bontempi, 2023).

From this perspective, the object of solidarity is no longer society understood as a self-contained human collectivity, but the socio-ecological conditions that make collective existence possible. Human participation consequently becomes analytically significant not because human actors alone sustain habitability, but because they possess the capacity to organise reflexively their own participation within the broader socio-ecological processes through which habitability is continuously composed and reproduced.

Terrestrial solidarity therefore does not replace the organisational functions reconstructed by Climate Justice, Climate Solidarity, Commons and Commoning, Political Ecology or Earth System Governance. Rather, it provides the sociological framework within which these dispersed organisational functions become intelligible as complementary dimensions of the collective organisation of participation in the co-production of habitability.

Terrestrial solidarity thus designates the organisational form through which human societies reflexively participate in the co-production of habitability. In this way, solidarity is reconstructed not as the consequence of interdependence, nor simply as a normative aspiration, but as the sociological concept through which the collective organisation of participation in the co-production of habitability becomes theoretically intelligible (Bontempi, 2023).

## 4 Methodological note

The theoretical framework developed above requires a methodological strategy consistent with its reconstructive purpose. Accordingly, this article adopts a strategy of theoretical reconstruction rather than comparative theory-building or empirical theory testing (Abbott, 2004; Bourdieu et al., 1991). Its objective is not to evaluate competing approaches to climate change or to establish the empirical validity of a new explanatory model. Instead, it reconstructs how different theoretical traditions progressively disclose complementary organisational dimensions of an emerging sociological problem and subsequently recomposes these dimensions within a common conceptual framework.

The five perspectives reviewed in the previous section—Climate Justice, Climate Solidarity, Commons and Commoning, Political Ecology, and Earth System Governance—are therefore not treated as competing paradigms. Each contributes to making visible a distinct organisational dimension of the changing relationship between collective existence and the material conditions upon which it depends. The methodological purpose of the proposed framework is not to synthesise these perspectives into a unified theory, but to reconstruct the theoretical operations through which their partial contributions can be understood as participating in a single sociological explanation.

This reconstructive strategy also shapes the role assigned to the empirical material. The Rhine River basin, the Danube River basin, and Community-Supported Agriculture are not introduced as cases selected to verify hypotheses, establish causal inference, or demonstrate the empirical superiority of the proposed framework. Nor are they presented as representative examples of terrestrial solidarity. Rather, they function as heterogeneous empirical configurations through which distinct theoretical operations become separately observable before being recomposed within a single sociological explanation.

The heuristic value of empirical heterogeneity lies precisely in this analytical separation. Because the three configurations differ substantially in their ecological dynamics, institutional arrangements, temporal horizons and organisational forms, they illuminate different dimensions of the theoretical framework with particular clarity. River-basin governance makes visible the collective organisation of distributed ecological processes across complex institutional configurations; Community-Supported Agriculture reveals the collective organisation of participation in the ongoing reproduction of socio-ecological conditions. Considered together, these heterogeneous configurations render the organisational dimensions of terrestrial solidarity observable without reducing them to a single empirical setting.

The relationship between theory and empirical material is therefore reconstructive rather than illustrative, demonstrative or verificatory. The theoretical framework provides the conceptual architecture through which the empirical configurations become intelligible, while the empirical configurations make the framework’s theoretical operations observable in concrete organisational settings. Their function is neither to confirm nor to exemplify the theory in a conventional sense, but to render analytically accessible the organisational processes through which terrestrial solidarity can be reconstructed as a sociological concept.

This methodological strategy differs both from comparative case analysis and from empirical theory testing. Comparative research typically seeks to explain variation across cases, whereas empirical theory testing evaluates the explanatory capacity of competing hypotheses. The present article pursues neither objective. Instead, it examines heterogeneous empirical configurations in order to render the framework’s theoretical operations observable under different organisational conditions. The cumulative reconstruction of these organisational operations subsequently provides the analytical bridge between the theoretical framework and the sociological discussion developed in the following section.

## 5 From theoretical reconstruction to empirical configurations

The preceding sections have progressively reconstructed terrestrial solidarity by addressing a sequence of interconnected analytical questions concerning habitability, participation, mediation, historical persistence, recursive reproduction, distributed coordination and collective organisation. The methodological strategy outlined above now requires asking how these theoretical operations can become empirically observable. The theoretical framework developed through this progression is not intended to provide a predictive model or a set of variables to be empirically tested. Rather, its purpose is to offer an interpretive framework capable of making visible dimensions of collective organisation that remain analytically fragmented when habitability, governance, ecological interdependence and solidarity are treated separately. Instead of explaining specific empirical outcomes, the framework identifies a sequence of analytical questions through which different organisational configurations can be interpreted.

The empirical configurations that follow are selected because they illuminate distinct organisational arrangements—from transboundary river governance to community-supported agriculture—through which human actors organise reflexive obligations towards sustaining habitability. Their analytical value lies not in their representativeness, but in their capacity to show how the proposed framework reorients sociological observation by making visible organisational processes that conventional understandings of solidarity are less well equipped to capture. The objective is therefore not to demonstrate the theory through empirical evidence, but to render its organisational operations observable across heterogeneous empirical configurations.

### 5.1 A common analytical question across empirical configurations

The empirical configurations are examined through a common analytical question derived from the theoretical framework developed in the previous sections: how do different forms of collective organisation participate in maintaining the material conditions upon which collective existence depends? If terrestrial solidarity is understood as the collective organisation of participation in the co-production of habitability, no single empirical configuration can exhaust its analytical complexity. Different organisational arrangements instead render different organisational operations particularly observable.

The three empirical configurations have consequently been selected because each renders one organisational operation of the proposed framework particularly visible. The Rhine renders observable how heterogeneous ecological processes become institutionally coordinated through monitoring, mediation and organisational feedback. The Danube renders observable the heterarchical coordination of socio-ecological processes across multiple institutional levels without relying upon a single organising centre. Community-supported agriculture, represented here through experiences such as Arvaia and the German SoLaWi network, renders observable how collective organisation recursively regenerates the material conditions upon which its own continuity depends through shared risk, long-term commitments and the continuous reorganisation of material interdependencies. Addressing the common analytical question across these heterogeneous settings makes it possible to reconstruct how these complementary organisational operations become intelligible as dimensions of terrestrial solidarity.

### 5.2 Making ecological processes organisable: the Rhine

River basin governance provides a particularly suitable empirical setting for examining how distributed ecological processes become objects of continuous collective organisation. The common analytical question developed above is not how the Rhine has been successfully governed, but how heterogeneous ecological dynamics become progressively rendered observable, interpretable and organisationally actionable across a plurality of institutions operating under different jurisdictions and responsibilities. From this perspective, the Rhine is approached not primarily as a river to be managed, but as a socio-ecological configuration through which one organisational operation of terrestrial solidarity becomes empirically reconstructible.

The International Commission for the Protection of the Rhine (ICPR) brings together the riparian states, the European Union and a broad network of scientific, technical and administrative organisations responsible for river-basin monitoring, water-quality assessment, flood-risk management, ecological restoration and the implementation of common environmental objectives (International Commission for the Protection of the Rhine, 2020; International Commission for the Protection of the Rhine, 2025). Its analytical significance within the proposed framework lies not simply in the plurality of participating organisations, but in the continual coordination of heterogeneous forms of scientific expertise, institutional authority, regulatory instruments and ecological observation that no single participant could produce independently. Rather than constituting a central authority governing a single ecological system, the Commission has progressively established a distributed organisational infrastructure through which hydrological observations, chemical monitoring, ecological indicators, scientific expertise, legal instruments and political negotiations become continuously connected. The significance of this arrangement lies less in the existence of a coordinating institution than in the continual production of organisational relations through which heterogeneous ecological processes become collectively actionable.

Monitoring performs a function that extends well beyond the production of environmental knowledge. Measurements, ecological indicators and scientific observations do not simply represent environmental conditions; they continually reorganise the relations through which participating organisations perceive, interpret and respond to ecological transformations. Their coordinative significance lies precisely in their capacity to translate ecological variation into organisational constraints requiring continual institutional adjustment. Mediation is therefore not simply the circulation of information between actors, but the ongoing transformation of heterogeneous ecological processes, scientific observations, regulatory instruments and institutional practices into configurations capable of collective coordination. Information acquires organisational significance only insofar as it becomes incorporated into recurrent processes of collective adjustment. Feedback, in this sense, should not be understood merely as an ecological property of the river system but as an organisational process through which the collective continually modifies its own modes of coordination.

Within the reconstructive framework developed above, this dynamic makes visible what has been defined as reflexive obligation. As participating organisations contribute to maintaining the ecological conditions upon which the river basin depends, they cannot remain organisationally unchanged when those conditions themselves are transformed. The obligation involved is therefore neither moral nor contractual. It emerges from participation itself. As long as the collective participates in producing the conditions of its own continuity, it cannot avoid reorganising itself when those conditions change. This organisational reconfiguration is constrained by historically sedimented institutional arrangements that open some pathways of transformation while closing others; it simultaneously participates in regenerating the material conditions upon which continued cooperation depends and redistributes capacities for action across the heterogeneous participants involved.

Rather than demonstrating the effectiveness of river-basin governance, the Rhine configuration renders one constitutive organisational operation of terrestrial solidarity particularly visible. It shows how distributed ecological processes become progressively transformed into enduring forms of collective organisation through continuous mediation, organisational feedback and institutional reconfiguration. In doing so, it renders analytically observable how ecological processes become collectively organisable without presupposing either centralised control or the reduction of ecological complexity.

### 5.3 Maintaining coordination across distributed temporalities: the Danube

River basin governance provides a particularly suitable empirical setting for examining how collective organisation can remain coherent when the ecological and institutional processes upon which it depends are distributed across multiple jurisdictions, organisational levels and temporal horizons. The common analytical question developed above is not how such a large river basin has been successfully governed, but how heterogeneous processes become capable of participating in a common organisational configuration without being subordinated to a single centre of authority. From this perspective, the Danube is approached not primarily as an example of transboundary water governance, but as an empirical configuration through which one organisational operation of terrestrial solidarity becomes empirically reconstructible.

The International Commission for the Protection of the Danube River (ICPDR) brings together 14 riparian states, the European Union, national environmental authorities, regional administrations, scientific organisations and basin-wide monitoring programmes within a shared framework for river-basin management (International Commission for the Protection of the Danube River, 2021; International Commission for the Protection of the Danube River, 2025). Joint management plans, basin-wide monitoring systems, flood-risk coordination, ecological restoration programmes and common implementation strategies provide the organisational infrastructure through which these heterogeneous participants coordinate their activities while preserving differentiated jurisdictions, competencies and responsibilities. Its analytical significance within the proposed framework lies in demonstrating how coordination emerges through the continuous articulation of multiple institutional levels rather than through hierarchical integration.

This organisational arrangement becomes particularly significant because the ecological, political and institutional processes involved do not evolve according to a common temporal rhythm. Hydrological dynamics, ecological restoration, legal reforms, infrastructural investments, monitoring programmes and political decision-making all operate according to partially independent and frequently asynchronous temporalities. Coordination therefore cannot consist simply in aligning simultaneous actions. Rather, it requires the continual composition of organisational relations capable of maintaining coherence across processes whose temporal horizons remain intrinsically plural.

Within the reconstructive framework developed above, this configuration renders visible the organisational significance of heterarchy. Heterarchy does not designate the absence of organisation, but the continuous coordination of heterogeneous capacities for action distributed across institutions, scales and temporalities that cannot be reduced to a single organising principle. The persistence of the collective consequently depends less upon centralised authority than upon the ongoing reorganisation of relations among partially autonomous participants whose capacities for intervention remain differentiated and continually evolving. Historicity acquires particular significance because previous institutional configurations continue to shape the range of future organisational possibilities. Earlier agreements, monitoring infrastructures, legal arrangements and accumulated forms of cooperation do not determine subsequent developments, but they sediment conditions that open some trajectories of coordination while rendering others less available.

Rather than demonstrating the effectiveness of multi-level governance, the Danube configuration renders one constitutive organisational operation of terrestrial solidarity particularly visible. It shows how distributed socio-ecological processes remain collectively coordinated through heterarchical organisational arrangements operating across multiple, asynchronous temporalities. In doing so, it renders analytically observable how collective organisation can maintain coherence across distributed temporalities without presupposing either institutional centralisation or temporal uniformity.

### 5.4 Regenerating material dependencies through collective organisation: community-supported agriculture

Community-supported agriculture provides a particularly suitable empirical setting for examining how collective organisation participates in regenerating the material conditions upon which its own continuity depends. The common analytical question developed above is not how cooperative farming is organised, but how material dependencies themselves become collectively organised through recurring forms of cooperation linking ecological processes, agricultural production and everyday social practices. From this perspective, community-supported agriculture is approached not primarily as an alternative food network, but as an empirical configuration through which one organisational operation of terrestrial solidarity becomes empirically reconstructible.

Community-supported agriculture brings together farmers, members, agricultural land, soils, crops, seasonal cycles, financial commitments and everyday work within a single organisational arrangement. Experiences such as Arvaia in Italy and the German SoLaWi network are based upon advance financial contributions, the collective sharing of production risks, the seasonal distribution of harvests and the continuous participation of members in sustaining agricultural activity (European CSA Research Group, 2016; URGENCI, 2025; Arvaia, 2025; Netzwerk Solidarische Landwirtschaft, 2025). Their analytical significance within the proposed framework lies not simply in the production of food through cooperative arrangements, but in the organisation of durable relations through which ecological processes and collective commitments become mutually constitutive. Rather than separating production, consumption and environmental stewardship into distinct domains, these organisational forms reconnect them within recurrent practices of collective maintenance.

Within the reconstructive framework developed above, the central organisational operation performed by these experiences extends beyond cooperation itself. Advance payments, shared risks, seasonal planning, soil management and collective decision-making do not simply facilitate agricultural production; they recursively reorganise the material dependencies upon which continued cooperation depends. Material dependence does not itself constitute an organisational condition. It becomes sociologically significant through the collective organisation of participation in the material dependencies upon which collective existence depends. The significance of these practices therefore lies in their capacity to transform dependence from an external constraint into a collectively maintained condition of continuity. Cooperation is consequently sustained not through the pursuit of shared preferences alone, but through the recurrent organisation of the material conditions that make cooperation itself possible.

This organisational configuration renders visible what has been defined above as autotrophy. Collective organisation does not merely adapt to pre-existing ecological conditions but participates in regenerating them through recursive processes linking ecological reproduction, agricultural work and collective commitments. The recursive character of this organisation does not imply organisational self-sufficiency nor complete closure of material cycles. Rather, it designates the capacity of the collective to participate continuously in regenerating the conditions upon which its own persistence depends. As these conditions evolve, organisational practices are repeatedly reconfigured, while historically sedimented arrangements simultaneously enable certain trajectories of adaptation and constrain others. The organisation of material dependencies therefore remains both historically situated and continuously transformable.

Rather than illustrating local cooperation or sustainable food production, the community-supported agriculture configuration renders one constitutive organisational operation of terrestrial solidarity particularly visible. It shows how collective organisation recursively regenerates the material dependencies upon which its own continuity depends through the organised participation of members in sustaining the conditions of collective existence. In doing so, it renders analytically observable how collective organisation participates in regenerating the material conditions upon which its own continuity depends through the continuous organisation of participation in material dependencies.

## 6 Discussion

### 6.1 Reconstructing the organisational architecture of terrestrial solidarity

The three empirical analyses presented above should not be understood as identifying different forms of terrestrial solidarity. Rather, they reconstruct three complementary organisational operations through which terrestrial solidarity becomes sociologically intelligible. The empirical configurations differ substantially in their institutional arrangements, ecological settings and organisational practices. Yet, when examined through the common analytical question developed in this article, they reveal complementary organisational processes participating in the collective organisation of the material conditions upon which collective existence depends.

Taken separately, each empirical configuration renders one organisational operation particularly observable. The Rhine reconstructs how heterogeneous ecological processes become collectively organisable through continuous mediation and organisational feedback. The Danube demonstrates how distributed socio-ecological processes remain collectively coordinated despite operating across multiple jurisdictions, organisational levels and asynchronous temporalities. Community-supported agriculture makes visible how collective organisation participates recursively in regenerating the material dependencies upon which its own continuity depends. None of these organisational operations, however, constitutes terrestrial solidarity in isolation. Each represents a necessary component of a broader organisational architecture.

Considered together, these organisational operations reveal that terrestrial solidarity cannot be reduced either to institutional coordination, cooperative commitment or environmental governance alone. The co-production of habitability requires the continuous composition of mediation, heterarchical coordination and recursive regeneration as mutually dependent organisational operations. Ecological processes become organisationally actionable through mediation; organisational coherence is maintained through heterarchical coordination across multiple jurisdictions, institutional levels and temporalities; and collective continuity depends upon the recursive regeneration of the material conditions that sustain it. These operations do not function as successive stages of a linear process. Rather, they continuously compose and reshape one another within the organisational architecture through which participation in the co-production of habitability becomes collectively organised.

The theoretical contribution of terrestrial solidarity therefore lies not in identifying a new empirical category of collective action, but in reconstructing these organisational operations within a single sociological explanation. Rather than treating mediation, coordination and regeneration as analytically separate processes, the proposed framework shows how they become interdependent components of the organisational architecture of habitability. The empirical configurations do not demonstrate the existence of terrestrial solidarity; they render analytically observable the organisational operations that the present framework subsequently recomposes into a single sociological explanation.

### 6.2 The contribution of terrestrial solidarity to contemporary sociological theory

The theoretical contribution of terrestrial solidarity does not lie in proposing an additional normative model of solidarity for the climate age. Rather, it lies in reconstructing the sociological concept of solidarity around a different explanatory horizon: not the reproduction of society alone, but the collective organisation of the co-production of habitability. The shift is not primarily normative but analytical. It does not replace existing understandings of solidarity with a new ethical ideal; instead, it redefines what the sociological concept of solidarity is called upon to explain under conditions in which the material conditions of collective existence have themselves become increasingly shaped by collective human activity.

From this perspective, the climate crisis does not simply introduce new objects into sociological analysis. It transforms the relationship between collective organisation and the conditions upon which collective existence depends. Classical theories generally understood solidarity as organising cooperation among social actors in order to reproduce social integration. Contemporary climate-related scholarship has progressively expanded this horizon by foregrounding differentiated responsibilities, durable commitments, common resource maintenance, socio-ecological production and multi-level coordination. Each of these perspectives has contributed essential analytical insights. Yet each reconstructs only one component of a broader organisational transformation that increasingly concerns the organisation of habitability itself.

The framework proposed here therefore seeks neither to replace nor to synthesise these perspectives in the conventional sense. Rather, it reconstructs the organisational relations through which their respective analytical contributions become mutually intelligible within a common explanatory architecture. Climate Justice renders visible the redistribution of responsibilities generated by differentiated vulnerabilities and capacities. Climate Solidarity demonstrates how durable commitments can stabilise collective responses to long-term environmental transformations. Commons and Commoning foreground the collective maintenance of shared conditions of reproduction. Political Ecology reveals the co-production of socio-ecological relations through heterogeneous human and non-human participation. Earth System Governance reconstructs the institutional coordination required when ecological processes exceed the capacities of individual political jurisdictions. Considered separately, these perspectives illuminate distinct organisational contributions. Considered together, they indicate the emergence of a transformed explanatory horizon that none of them fully reconstructs independently.

Within this reconstructed explanatory horizon, dependence becomes analytically prior to cooperation. Cooperation remains indispensable, but it is no longer treated as the primary condition from which solidarity originates. Rather, cooperative practices appear as one possible organisational response to material dependencies that increasingly participate in shaping the conditions of collective existence. The analytical centre of gravity consequently shifts from cooperation itself towards the collective organisation of dependence. Dependence is not itself an organisational condition. Rather, it is the existential condition that renders the collective organisation of participation in the co-production of habitability sociologically meaningful. The question therefore becomes not simply how cooperation is achieved, but how collective organisation participates in organising, stabilising and transforming the material dependencies upon which collective existence depends.

This reformulation also repositions the sociological understanding of agency. Rather than treating agency as a pre-existing capacity possessed by individual or collective actors, terrestrial solidarity reconstructs it as a relational configuration continuously redistributed through the organisation of material dependencies. As collectives participate in producing the conditions of their own continuity, organisational reconfigurations simultaneously transform the distribution of capacities for intervention, responsibility and coordination among heterogeneous participants. Agency therefore appears less as the point of departure of collective action than as one of its continuously emerging organisational effects. This reformulation does not dissolve the distinction between social and ecological processes. Rather, it reconstructs their relation through the organisational arrangements by which heterogeneous human and non-human processes become collectively coordinated, recursively regenerated and historically transformed.

From this perspective, solidarity is no longer defined primarily by the integration of social actors, but by the collective organisation of participation in the co-production of habitability.

In this sense, terrestrial solidarity is proposed not as a new empirical category of collective action, but as a sociological framework capable of reconstructing how contemporary forms of collective organisation increasingly participate in the co-production of habitability (Latour, 2017).

### 6.3 Theoretical recomposition and the transformation of the explanandum

The need for theoretical recomposition does not arise because existing frameworks are incomplete. It arises because each reconstructs organisational contributions in relation to a different explanandum. Climate Justice, Climate Solidarity, Commons and Commoning, Political Ecology and Earth System Governance each emerged to address distinct theoretical questions and therefore illuminate different organisational dimensions of collective existence under conditions of climate change. Their analytical differences should not be understood as competing explanations of the same phenomenon, but as consequences of the different problems they seek to explain. Once the object of sociological explanation shifts from the reproduction of society alone to the collective organisation of the co-production of habitability, these organisational contributions can no longer remain analytically separate.

The originality of terrestrial solidarity lies neither in replacing these perspectives nor in integrating them into a more comprehensive model. Rather, it lies in reconstructing the organisational relations through which their distinct theoretical contributions become mutually dependent within a common sociological explanation. Their analytical recomposition follows directly from the transformation of the explanandum: once habitability becomes the common explanatory horizon, organisational processes previously reconstructed in relation to distinct theoretical problems become intelligible as interdependent dimensions of the collective organisation of participation in its co-production.

Seen from this perspective, terrestrial solidarity does not propose an additional theory alongside existing approaches. It reconstructs the organisational architecture through which insights developed across different theoretical traditions become intelligible as components of a common explanatory framework. The contribution of the present article therefore lies less in introducing new empirical phenomena than in making visible the organisational coherence linking processes that have largely been theorised separately because they emerged within different explanatory traditions.

This reformulation also carries broader implications for sociological theory. It suggests that the climate crisis should not be understood merely as a new empirical domain requiring additional concepts, but as a transformation of the explanandum around which the sociological concept of solidarity has historically been organised. When collective existence increasingly depends upon the organised participation of heterogeneous human and non-human processes in the co-production of habitability, solidarity can no longer be reconstructed exclusively as a mechanism of social integration or cooperative coordination. It becomes the sociological concept through which the collective organisation of participation in the co-production of habitability becomes theoretically intelligible.

### 6.4 Terrestrial solidarity as a sociological concept

The proposed framework should not be understood as extending sociology towards Earth System Science, nor as translating ecological processes into sociological language. Its purpose is fundamentally different. Terrestrial solidarity remains a sociological concept because its primary object is neither ecological dynamics nor environmental governance as such, but the forms of collective organisation through which heterogeneous participants organise their participation in the co-production of the conditions of collective existence. Ecological processes become sociologically relevant not because they replace social relations as explanatory objects, but because they increasingly enter into the organisational configurations through which collective existence is organised, reproduced and transformed.

From this perspective, terrestrial solidarity does not dissolve the distinction between the social and the ecological. Rather, it reconstructs their relationship through the organisation of participation. What sociology seeks to explain is therefore not ecological change itself, but the collective arrangements through which ecological transformations become organisationally consequential. The analytical focus remains firmly sociological: institutions, reflexive obligations, coordination, organisational reconfiguration, historical trajectories and the redistribution of capacities for action. What changes is not sociology’s object of inquiry, but the explanatory horizon within which these processes acquire sociological significance.

Consequently, terrestrial solidarity should not be understood as a theory of environmental governance alone. It is a sociological framework for explaining how collective organisation increasingly participates in shaping, maintaining and transforming the material conditions upon which collective existence depends. The climate crisis therefore does not move sociology beyond its own domain; rather, it reconfigures the explanatory horizon through which sociology reconstructs the organisation of collective existence.

## 7 Conclusion

This article has argued that climate change transforms not simply the empirical context within which solidarity operates, but the sociological explanandum that solidarity has traditionally been called upon to explain. Classical sociology reconstructed solidarity as the organisational principle through which differentiated societies maintained their continuity over time. Contemporary climate-related scholarship has progressively expanded this explanatory horizon by foregrounding responsibility, durable commitments, common maintenance, socio-ecological co-production and institutional coordination. Yet these organisational contributions have largely remained distributed across distinct theoretical traditions because they emerged in response to different explanatory problems.

The concept of terrestrial solidarity proposed here seeks to reconstruct these organisational contributions within a common sociological framework centred on the collective organisation of the co-production of habitability. Rather than introducing a further normative model of solidarity, the article has argued that the transformation of the explanandum requires a corresponding reconstruction of solidarity itself. From this perspective, solidarity is no longer defined primarily by the integration of social actors, but by the collective organisation of participation in the co-production of habitability.

A second implication concerns the sociological status of dependence. Classical sociology understood increasing interdependence as compatible with the progressive emancipation of individuals from inherited forms of dependence. As the division of labour expanded, individuals became increasingly dependent upon one another while simultaneously acquiring greater autonomy from traditional social constraints. Within the Critical Zone, however, dependence assumes a different analytical status. It no longer refers primarily to functional interdependence among differentiated social actors, but to the constitutive participation of heterogeneous human and non-human processes in sustaining the conditions of collective existence. These dependencies may be organised, transformed, mediated and unequally distributed, but they cannot be overcome through emancipation from the material conditions of life themselves.

Terrestrial solidarity therefore implies a corresponding reconstruction of freedom. Freedom no longer appears primarily as independence from constitutive dependencies, but as the collective capacity to organise, stabilise and transform the relations of dependence through which habitability is continuously co-produced. The recognition of constitutive limits does not diminish human agency; rather, it relocates agency within the organisational processes through which collective existence becomes materially sustainable. In this sense, terrestrial solidarity resonates with recent attempts to rethink freedom not beyond the conditions of terrestrial existence, but through the continuous composition of the heterogeneous relations that make collective existence possible.

The argument developed here remains intentionally reconstructive. It does not claim to provide a comprehensive theory of socio-ecological transformation, nor to resolve the normative and political controversies surrounding climate action. Rather, it proposes a sociological framework capable of reconstructing the organisational architecture through which participation in the co-production of habitability becomes collectively organised. Future research may extend this framework by exploring how different institutional arrangements, forms of reflexive organisation and historically sedimented dependencies reshape the conditions under which terrestrial solidarity emerges across different socio-ecological contexts.

If climate change transforms the explanandum of solidarity, sociology requires conceptual resources capable of reconstructing the organisational arrangements through which collective participation in the co-production of habitability becomes possible. Terrestrial solidarity is proposed here not as a replacement for existing theories of solidarity, but as a sociological reconstruction of solidarity for the terrestrial condition, where dependence, freedom and collective organisation become mutually constitutive dimensions of the co-production of habitability.

## Data Availability

The original contributions presented in the study are included in the article/supplementary material, further inquiries can be directed to the corresponding author.
